# Novel microtubule inhibitor MPT0B098 inhibits hypoxia-induced epithelial-to-mesenchymal transition in head and neck squamous cell carcinoma

**DOI:** 10.1186/s12929-018-0432-6

**Published:** 2018-03-28

**Authors:** I-Ting Tsai, Ching-Chuan Kuo, Jing-Ping Liou, Jang-Yang Chang

**Affiliations:** 1 National Institute of Cancer Research, National Health Research Institutes, Tainan, Taiwan; 2 Institute of Molecular Medicine, College of Medicine, National Cheng Kung University, Tainan, Taiwan; 3 Institute of Biotechnology and Pharmaceutical Research, National Health Research Institutes, Zhunan, Taiwan; 4 Institute of Clinical Pharmacy and Pharmaceutical Sciences, College of Medicine, National Cheng Kung University, Tainan, Taiwan; 5 Graduate Program for Aging, China Medical University, Taichung, Taiwan; 6 College of Pharmacy, Taipei Medical University, Taipei, Taiwan; 7 Division of Hematology/Oncology, Department of Internal Medicine, National Cheng Kung University Hospital, College of Medicine, National Cheng Kung University, Tainan, Taiwan

**Keywords:** Hypoxia, Epithelial to mesenchymal transition, Microtubule inhibitor, TGF-β, Head and neck cancer

## Abstract

**Background:**

Tumor hypoxia-induced epithelial–mesenchymal transition (EMT) is critical in promoting cancer metastasis. We recently discovered a novel microtubule inhibitor, MPT0B098, that employs a novel antitumor mechanism. It destabilizes hypoxia-inducible factor (HIF)-1α mRNA by blocking the function of human antigen R. Thus, we proposed that MPT0B098 modulates hypoxia-induced EMT.

**Methods:**

In vitro IC_50_ values were determined through the methylene blue dye assay. To investigate molecular events, reverse transcriptase-polymerase chain reaction, Western blotting, immunofluorescence staining, and wound healing assay were employed.

**Results:**

MPT0B098 significantly inhibited HIF-1α expression, epithelial-to-mesenchymal morphology changes, and migratory ability in the human head and neck squamous cell carcinoma cell line OEC-M1. Furthermore, after MPT0B098 treatment, the expression of two mesenchymal markers, vimentin and N-cadherin, was downregulated under hypoxic conditions. Moreover, MPT0B098 suppressed hypoxia-induced EMT in part by inhibiting EMT-activating transcription factors, Twist and SNAI2/Slug. In addition, the inhibition of hypoxia-induced F-actin rearrangement and focal adhesion kinase phosphorylation may have contributed to suppression of EMT by MPT0B098in OEC-M1 cells. MPT0B098 significantly inhibited transforming growth factor(TGF)-β-induced phosphorylation of receptor-associated Smad2/3 by downregulating TGF-β mRNA and protein expression.

**Conclusions:**

Taken together, this study provides a novel insight into the role of MPT0B098 in inhibiting hypoxia-induced EMT, suggesting its potential use for treating head and neck cancers.

**Electronic supplementary material:**

The online version of this article (10.1186/s12929-018-0432-6) contains supplementary material, which is available to authorized users.

## Background

In solid tumors, blood vessels form abnormally and are dysfunctional, resulting in an inability to supply sufficient oxygen and nutrients to the growing tumor mass [1]. Although a decrease in oxygen tension can be lethal for some cells, many tumor cells can survive under hypoxic conditions [2]. Tumor cells in hypoxia are resistant to radiation and chemotherapy; furthermore, hypoxic conditions can promote tumor progression and metastasis through numerous direct and indirect mechanisms [2]. The most critical factor responding to hypoxic conditions is hypoxia-inducible factor (HIF)-1α, a basic helix–loop–helix transcription factor composed of an α subunit (regulated by the amount of oxygen tension) and β subunit (constitutively expressed) [3].

Epithelial–mesenchymal transition (EMT) is a cellular program in which epithelial cells lose their cell polarity and cell–cell adhesion ability and become mesenchymal cells by acquiring a fibroblastic morphology and migratory and invasive features. First recognized as a characteristic of embryogenic development, EMT is now indicated to be critical in tumor malignant transformation and metastasis [4]. Ample evidence indicates that hypoxia leads to characteristic changes in cell morphology that induce a mesenchymal-like phenotype and facilitate tumor cell metastasis [5].

**Fig. 1:**
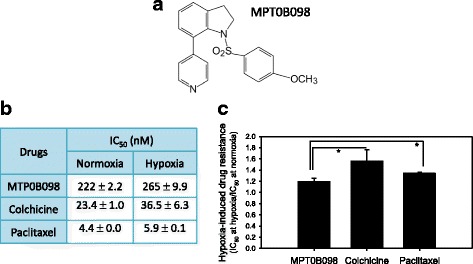
Antiproliferative effect of MPT0B098 in OEC-M1 cells under normoxic and hypoxic conditions. **a** The chemical structure of MPT0B098. **b** The in vitro antiproliferative activity of MPT0B098 in OEC-M1 cells under normoxic and hypoxic conditions. OEC-M1 cells were treated with MPT0B098, colchicine, or paclitaxel under normoxic and hypoxic conditions for 72 h. The IC_50_ values of these compounds resulting from 50% inhibition of cell growth were calculated using the methylene blue dye assay. Each value represents the mean ± SD of three independent experiments. **c** Hypoxia-induced drug resistance is the IC_50_ value of the test compounds in hypoxia divided by the equivalent in normoxia (* *p* < 0.05)

A hypoxic microenvironment is frequently found in head and neck squamous cell carcinoma (HNSCC) and is known as a risk factor for prognosis [6, 7]. In addition, acute hypoxia can cause the development of aggressive cancers with high metastatic characteristics, resistance to chemotherapy, and higher tumor recurrence rates in patients with head and neck cancer [6, 8, 9]. We recently discovered a novel indoline-sulfonamide compound, 7-aryl-indoline-1-benzene-sulfonamide (MPT0B098; Fig. 1a), that is a potent microtubule inhibitor and effective against a panel of human cancer cell lines [10]. In contrast to other clinically used microtubule inhibitors, MPT0B098 is effective in suppressing tumor growth despite p-gp170/MDR status [11], and can destabilize HIF-1α mRNA under hypoxic conditions by inhibiting the translocation of human antigen R (HuR) from the nucleus to the cytoplasm [12]. On the basis of the ability of MPT0B098 to modulate HIF-1α, we proposed that this compound can modulate hypoxia-induced EMT in head and neck cancers. Thus, this study investigated the effects and underlying mechanisms of MPT0B098 on hypoxia-induced EMT in the highly invasive HNSCC cell line OEC-M1.

## Methods

### Chemicals and antibodies

MPT0B098 was synthesized by Prof. Jing-Ping Liou at the College of Pharmacy, Taipei Medical University, Taipei, Taiwan. The detailed synthetic procedure was described previously [11]. Colchicine and paclitaxel were purchased from Sigma-Aldrich (St. Louis, MO). Monoclonal antibodies for Smad2, *phospho*-Smad2 (Ser465/467), Smad3, *phospho*-Smad3, Smad2/3, and SNAI2/Slug and polyclonal antibodies for transforming growth factor (TGF)-β, focal adhesion kinase (FAK), and *phospho*-FAK were purchased from Cell Signaling Technology (Danvers, MA). Monoclonal antibodies for HIF-1α and N-cadherin were purchased from BD Biosciences (San Jose, CA). A polyclonal antibody for vimentin was purchased from Santa Cruz Biotechnology (Santa Cruz, CA). A polyclonal antibody for Twist was purchased from GeneTex (Irvine, CA). A monoclonal antibody for GAPDH was purchased from Merck Millipore (Darmstadt, Germany). All other chemicals of standard analytic grade or higher were purchased from E. Merck Co. (Darmstadt, Germany) or Sigma-Aldrich (St. Louis, MO).

### Cell lines and cell culture

OEC-M1 cells, derived from a human oral epidermoid carcinoma, were established and provided by Dr. Ching-Liang Meng from the Department of Dentistry, Tri-Service General Hospital, National Defense Medical Center, Taipei, Taiwan [13]. The cells were cultured in RPMI 1640 medium, supplemented with 10% fetal bovine serum in a humidified 5% CO_2_ incubator at 37 °C. For hypoxic conditions, cells were incubated in a hypoxic chamber with a gas mixture of 1% O_2_ and 5% CO_2_ balanced with nitrogen.

### Cell morphology assay

Cells were stained with 0.5% crystal violet in 95% ethanol for 1 h and then examined through light microscopy.

### Cell viability assay

Cells in a logarithmic growth phase were cultured at a density of 10,000 cells per well in a 24-well plate. The cells were exposed to various concentrations of the test drug for 72 h. The methylene blue dye assay [14] was used to evaluate drug effect on cell growth. The IC_50_ values resulting from 50% inhibition of cell growth were calculated graphically, compared with control group growth.

### Western blotting

Cells were initially seeded at a density of 4 × 10^5^ cells in 100-mm^2^ dishes. Following treatment, cell pellets were collected and lysed in a buffer (50 mM Tris-HCl, pH 8.0, 150 mM NaCl, 5 mM EDTA, 2 mM dithiothreitol, 2 mM Na_3_VO_4_, 0.25 mM PMSF, 10 mM NaF, 0.5% NP-40, and 20 μg/mL each of aprotinin, leupeptin, and pepstatin [proteinase inhibitors]). The supernatants were collected and the amount of protein was quantified. Equal amounts of protein from each lysate were separated by SDS-PAGE, blotted on polyvinylidenedifluoride membranes, conjugated with various specific primary antibodies, and then probed with appropriate secondary antibodies. The immunoreactive bands were detected using the enhanced chemiluminescent method and then visualized on Kodak Bio-MAX MR film.

### Wound healing assay

Cells were grown to 80% confluence on culture dishes; then, a wound was created in the center of the cell monolayer with a plastic pipette tip. The migration of cells into the wound area was assessed after 4, 6, 8, and 18 h at 37 °C under normoxic (5% CO_2_, 21% O_2_) and hypoxic (5% CO_2_, 1% O_2_, balanced with N_2_) conditions. The area of denuded surface was quantified immediately and at the time points of 4, 6, 8 h after wounding. The extent of wound closure was determined by calculating the ratio between the surface area of the wound for each time point and the surface of the initial wound.

### Immunofluorescence analysis of F-actin

OEC-M1 cells were fixed with 4% paraformaldehyde (Electron Microscopy Sciences, Hatfield, PA), permeabilized with 0.5% Triton X-100 in phosphate-buffered saline, treated with 0.1% sodium borohydride (Sigma-Aldrich, St. Louis, MO), blocked with 5% horse serum, and then incubated with phalloidin (Life Technologies, Gaithersburg, MD) for 30 min at room temperature in the dark. Nuclei were stained with DAPI. Cells were observed with an OLYMPUS fluorescence microscope.

### Reverse transcriptase-polymerase chain reaction analysis

Ten micrograms of total RNA, extracted with Trizol reagent (Life Technologies, Gaithersburg, MD), were treated with DNase and converted to cDNA using the SuperScript II RNase H Reverse Transcriptase System (Invitrogen, Carlsbad, CA). Reverse transcriptase-polymerase chain reaction (RT-PCR) was performed using a Perkin-Elmer GeneAmp PCR System 2400 (Applied Biosystems, Foster City, CA). PCR primers and TaqMan probes (5′-GACCTGGA-3′) used to amplify the indicated genes were designed using Primer Express (version 1.0; Applied Biosystems) as follows: TGF-β1 forward 5′-cggagttgtgcggcagtggttga-3′ and reverse 5′-ggcgcccgggttatgctggttgta-3′ and TGF-β2 forward 5′-gtcttggatgcggcctattgc-3′ and reverse 5′-gctgcatttgcaagactttac-3′. The reaction mixture was preheated at 95 °C for 5 min, followed by 30 cycles of 95 °C for 30 s, 55 °C for 30 s, and 72 °C for 40 s; final extension was at 72 °C for 7 min. In a separate reaction, GAPDH was amplified as the reference gene. PCR products of the target genes were analyzed through electrophoresis on 2% agarose gel and visualized using ethidium bromide staining under UV light. Quantitative data was collected by measuring the bands using ImageJ (Bethesda, MD).

### Statistical analysis

Quantitative data were reported as mean ± SD. Statistical calculations were completed on Microsoft Excel. *P* values for determining statistical significance were calculated using an unpaired two-tailed Student’s *t* test.

## Results

### MPT0B098 exhibits low-level resistance toward OEC-M1 cell growth under hypoxic conditions

We used the methylene blue dye assay to examine the antiproliferative efficacy of MPT0B098 and other clinically used microtubule inhibitors, such as colchicine and paclitaxel, in OEC-M1 cells. As shown in Fig. 1b, MPT0B098 inhibited the growth of OEC-M1 cells with IC_50_ of 222 and 265 nM under normoxic and hypoxic conditions, respectively. This result indicates that hypoxia leads to increased low-level drug resistance of MPT0B098 in OEC-M1 cells (Fig. 1c).

In addition, compared with MPT0B098, other microtubule inhibitors, including colchicine and paclitaxel, exhibited higher resistance in OEC-M1 cells under hypoxic conditions than under normoxic conditions. The IC_50_ values of colchicine were 23 and 37 nM under normoxia and hypoxia, respectively, and the IC_50_ values of paclitaxel were 4.4 and 5.9 nM, respectively (Fig. 1b). These results indicate that MPT0B098 is more effective in overcoming hypoxia-induced drug resistance than colchicine and paclitaxel in OEC-M1 cells.

### MPT0B098 inhibits hypoxia-induced EMT in OEC-M1 cells

**Fig. 2:**
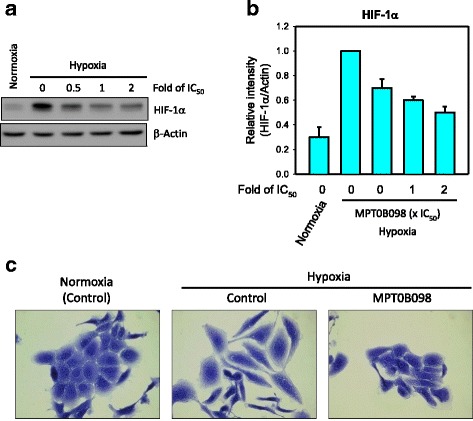
MPT0B098 inhibits hypoxia-induced EMT in OEC-M1 cells. **a** The effect of MPT0B098 onhypoxia-induced HIF-1αexpression. OEC-M1 cells were treated with various concentrations, indicated as fold of IC_50_ values, of MPT0B098 for 18 h under hypoxic conditions. At the end of the drug treatment, cell lysates were prepared and analyzed by SDS-PAGE and Western blot. β-Actin was used as an internal control. **b** Each bar depicts the mean of the relative intensity of HIF-1α from three independent experiments. **c** The effect of MPT0B098 on hypoxia-induced EMT.Cells were treated with MPT0B098 at a concentration of 0.5-fold IC_50_ for 48 h under hypoxic conditions and then cell morphology was examined by crystal violet staining. Cells in normoxia were used as controls

Intratumoral hypoxia induces EMT and promotes cancer metastasis. HIF-1α plays a critical role in driving the characteristic changes in cell morphology causing a mesenchymal-like phenotype and facilitating the metastasis of tumor cells [5, 15]. Because MPT0B098 can inhibit HIF-1α mRNA and protein expression in the human lung adenocarcinoma cell line A549 [12], we speculated that this compound inhibits HIF-1α expression and suppresses EMT in OEC-M1 cells. Consistent with our previous findings, MPT0B098 demonstrated potent inhibition of HIF-1α expression in a concentration-dependent manner under hypoxic conditions in OEC-M1 cells (Fig. 2a and b)**.** In addition, the inhibitory effect of MPT0B098 on HIF-1α was found in another human HNSCC cell line, SCC-15 (Additional file 1: Figure S1).

On further examining the role of MPT0B098 in hypoxia-induced EMT in OEC-M1 cells, we found that OEC-M1 cells displayed epithelial characteristics under normoxic conditions, with a round morphology and linked cells (Fig. 2c, *left panel*). However, under hypoxic conditions, cells displayed a fibroblastic morphology and lost their cell–cell contact, which is a mesenchymal characteristic, implying that hypoxia can trigger EMT (Fig. 2c, *middle panel*). Notably, the transformation from epithelial to mesenchymal cell type under hypoxic conditions could be inhibited by treating cells with MPT0B098 (Fig. 2c, *right panel*). These results suggest that MPT0B098 may play a role in modulating hypoxia-induced EMT in OEC-M1 cells.

### MPT0B098 is more potent in inhibiting hypoxia-induced mesenchymal marker expression than clinically used microtubule inhibitors

**Fig. 3:**
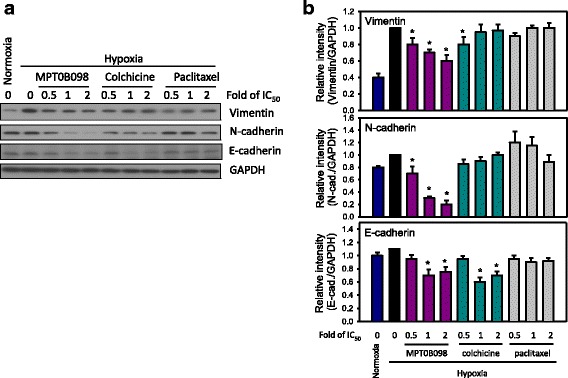
Effect of MPT0B098 and other microtubule inhibitors on the expression of EMT-related proteins in OEC-M1 cells under hypoxic conditions. **a** Parallel comparison of the effects of MPT0B098, colchicine, and paclitaxel on the expression of vimentin, N-cadherin, and E-cadherin in OEC-M1 cells.OEC-M1 cells were treated with various concentrations,indicated as fold of IC_50_ values,of MPT0B098, colchicine, or paclitaxel for 36 h under hypoxic conditions. At the end of the drug treatment, cell lysates were prepared and analyzed by SDS-PAGE and Western blot. GAPDH was used as an internal control. **b** Each bar depicts the mean of the relative intensity of vimentin, N-cadherin, and E-cadherin from three independent experiments (* *p* < 0.05, compared to hypoxia control).

MPT0B098 could inhibit the expression of hypoxia-induced mesenchymal markers, including vimentin and N-cadherin, in a concentration-dependent manner in OEC-M1 cells (Fig. 3a and 3b). Because MPT0B098 inhibited hypoxia-induced EMT, we analyzed whether other clinically used microtubule inhibitors, such as colchicine and paclitaxel, also suppress vimentin and N-cadherin expression in OEC-M1 cells under hypoxic conditions. MPT0B098 caused a reduction in vimentin and N-cadherin expression in a concentration dependent manner, but the same effect was not observed in colchicine- or paclitaxel-treated cells (Fig. 3a and 3b).

### MPT0B098 suppresses hypoxia-induced EMT partially by inhibiting the expression of EMT-activating transcription factors Twist and SNAI2/slug

**Fig. 4:**
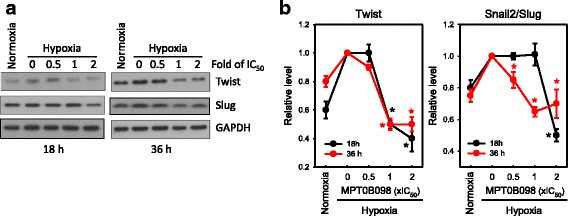
MPT0B098 suppresses hypoxia-induced EMT by inhibiting the expression of EMT-activating transcription factors, Twist and SNAI2/Slug. **a** The effects of MPT0B098 on the expression of Twist and SNAI2/Slug in OEC-M1 cells.OEC-M1 cells were treated with various concentrations, indicated as fold of IC_50_ values, of MPT0B098 for 18 and 36 h under hypoxic conditions. At the end of the drug treatment, cell lysates were prepared and analyzed by SDS-PAGE and Western blot. GAPDH was used as an internal control. Each values depicts the mean of the relative intensities of Twist and SNAI2/Slug from three independent experiments. **b** Each value depicts the mean of the relative intensities of Twist and SNAI2/Slug from three independent experiments (* *p* < 0.05, compared to hypoxia control)

During EMT, molecular reprogramming is triggered and orchestrated by various EMT-activating transcription factors, including Twist and SNAI2/Slug [16]. As shown in Fig. 4a and b, we found that the expression levels of Twist and SNAI2/Slug were significantly suppressed in a concentration-dependent manner when cells were treated with various concentrations of MPT0B098 under hypoxic conditions for 18 and 36 h. These results indicate that MPT0B098 suppresses hypoxia-induced EMT partially by inhibiting the expression of EMT-activating transcription factors, Twist and SNAI2/Slug, in OEC-M1 cells.

### MPT0B098 significantly inhibits hypoxia-induced F-actin rearrangement and FAK phosphorylation

**Fig. 5:**
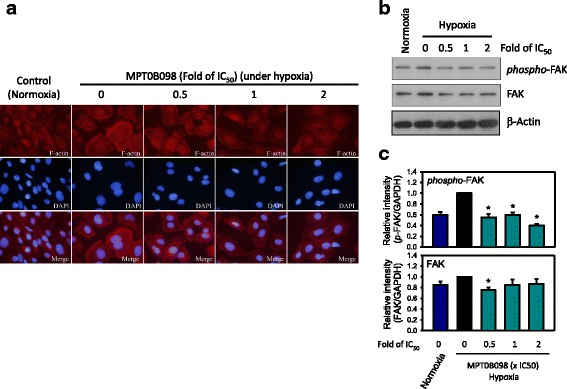
MPT0B098 inhibits hypoxia-induced F-actin rearrangement and FAK phosphorylation. **a** Immunofluorescent analysis of F-actin. OEC-M1 cells were treated with various concentrations, indicated as fold of IC_50_, of MPT0B098 for 18 h under hypoxic conditions, stained with phalloidin to label F-actin (red), counterstained with DAPI (blue), and then observed using an OLYMPUS florescence microscope. **b** Effect of MPT0B098 on FAK phosphorylation and expression. OEC-M1 cells were treated with various concentrations of MPT0B098 for 18 h.At the end of the drug treatment, cell lysates were prepared and analyzed by SDS-PAGE and Western blot. β-Actin was used as an internal control. **c** Each bar depicts the mean of the relative intensities of Twist and SNAI2/Slug from three independent experiments (* p < 0.05, compared to hypoxia control)

Actin filaments (F-actin) are organized in thin bundles in epithelial cells; however, in trans-differentiated mesenchymal cells, they are bundled into thick contractile stress fibers at the cell surface. Thus, F-actin rearrangement is associated with increased cell movement during EMT; it is also required for metastatic cancer cells to spread from primary tumors [17, 18]. As shown in Fig. 5a, f-actin is located within the cytoplasm under normoxic conditions. By contrast, hypoxia-induced F-actin rearrangement showing increased expression of stress fiber patterns and concentrated at the plasma membrane on the edge of the cells. MPT0B098-treated OEC-M1 cells demonstrated a reduction in the expression of hypoxia-induced stress fiber patterns and membrane localization of F-actin in a concentration-dependent manner.

FAK is a cytoplasmic tyrosine kinase, with critical roles in the regulation of cell spreading, migration, and survival and in the downstream signaling cascade associated with its own phosphorylation [19, 20]. Changes in the organization of the actin cytoskeleton, influenced by FAK, leads to remarkable changes in the tyrosine phosphorylation of several signaling proteins localized at the focal adhesion complex [21]. To further explore the molecular mechanisms responsible for MPT0B098 inhibition of hypoxia-induced actin cytoskeletal rearrangement, we examined the expression and activation of FAK protein. As shown in Fig. 5b and c, FAK expression and activation were increased under hypoxic conditions. Hypoxia-induced phosphorylation of FAK decreased when the hypoxic cells were treated with MPT0B098. Taken together, these results suggest that MPT0B098-inhibited FAK/actin cytoskeletal rearrangement partially contributes to EMT suppression.

### MPT0B098 downregulates TGF-β-induced phosphorylation of receptor-associated Smads

**Fig. 6:**
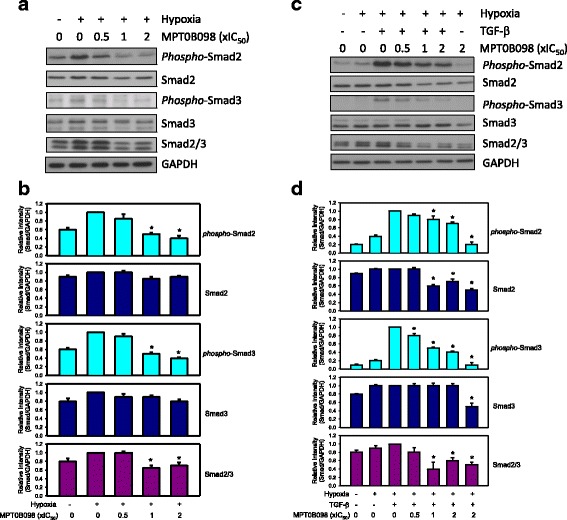
MPT0B098 downregulatesTGF-β/Smad signalingin OEC-M1 cells. **a** Cells were treated with various concentrations,indicated as fold of IC_50_ values, of MPT0B098 for 36 h under hypoxic conditions. **b** Each bar depicts the mean of the relative intensities of *phospho*-Smads and Smads from three independent experiments (* p < 0.05, compared to hypoxia control). **c** Cells were treated with various concentrations,indicated as fold of IC_50_ values, of MPT0B098 for 18 h, followed by an addition of 5 ng/mL TGF-β for another 18 h under hypoxic conditions. At the end of the drug treatment, cell lysates were prepared and analyzed by SDS-PAGE and Western blot. GAPDH was used as an internal control. **d** Each bar depicts the mean of the relative intensities of *phospho*-Smads and Smads from three independent experiments (* p < 0.05, compared to hypoxia control)

The reprogramming of gene expression during EMT is initiated and controlled by signaling pathways responding to extracellular cues, including TGF-β, fibroblast growth factor, epidermal growth factor, hepatocyte growth factor, Wnt/β-catenin, and Notch [22]. Among these, TGF-β signaling has a dominant role in the initiation of EMT programs that develop cancer progression [16, 22, 23]. We explored whether the TGF-β signaling pathway is involved in MPT0B098-mediated inhibition of hypoxia-induced EMT. Our results indicated that MPT0B098 suppressed the phosphorylation of Smad2 and Smad3 in hypoxia in OEC-M1 cells (Fig. 6a and b). In addition, MPT0B098 inhibited Smad signaling in another human HNSCC cell line, SCC-15 (Additional file 1: Figure S1).

To further examine the involvement of TGF-β in the hypoxia-induced activation of Smad signaling, TGF-β was applied to MPT0B098-treated hypoxic cells. TGF-β treatment significantly enhanced Smad2 and Smad3 phosphorylation even under hypoxic conditions (Fig. 6c and d). Coadministration of TGF-β and MPT0B098 significantly suppressed Smad2 and Smad3 phosphorylation in a concentration-dependent manner. In addition, MPT0B098 slightly inhibited Smad2 and Smad3 protein expression (Fig. 6c and d), suggesting that MPT0B098 influenced Smad inactivation under hypoxic conditions through TGF-β modulation.

### MPT0B098 downregulates TGF-β signaling by decreasing expression levels of TGF-β mRNA and protein

**Fig. 7:**
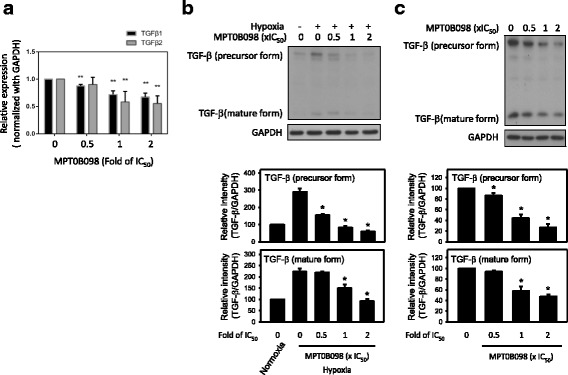
MPT0B098 downregulates TGF-β signaling by decreasing the expression of TGF-β mRNA and protein in OEC-M1 cells under hypoxic conditions. **a** Effect of MPT0B098 on the expression levels of TGF-β1 and TGF-β2 mRNA. Cells were treated with various concentrations of MPT0B098 in hypoxia. After incubation for 36 h, total RNA was extracted, reverse transcribed into cDNA, and subjected to PCR for detection of TGF-β1 and TGF-β2. GAPDH was used as an internal control. Data are represented as mean ± SD in triplicate. **P < 0.01 and ***P < 0.001for comparison between the control and treatment groups using an unpaired two-tailed Student’s t test. **b** Effect of MPT0B098 on the expression level of TGF-β protein. Cells were treated with various concentrations of MPT0B098 for 36 h in hypoxia. Quantification of TGF-β protein was determined by normalization with GAPDH (* *p* < 0.05, compared to hypoxia control). **c** The inhibitory effect of MPT0B098 on TGF-β under normoxic conditions. Cells were treated with MPT0B098 in normoxia at the indicated concentrations for 36 h. Quantification of TGF-β protein was determined by normalization with GAPDH (* *p* < 0.05, compared to control)

To further explore the relationship between MPT0B098 and TGF-β signaling, we examined the expression levels of TGF-β mRNA and protein in MPT0B098-treated hypoxic cells. We found that MPT0B098 suppressed TGF-β mRNA (Fig. 7a) and protein expression (Fig. 7b) in a dose-dependent manner under hypoxic conditions in OEC-M1 cells. In addition, we found that MPT0B098 inhibited TGF-β protein expression under normoxic conditions (Fig. 7c). These results suggest that MPT0B098 regulates the EMT process in part through a reduction in TGF-β mRNA and protein levels followed by downregulation of the TGF-β/Smad signaling cascades.

### MPT0B098 inhibits hypoxia-induced cell migration in OEC-M1 cells

**Fig. 8:**
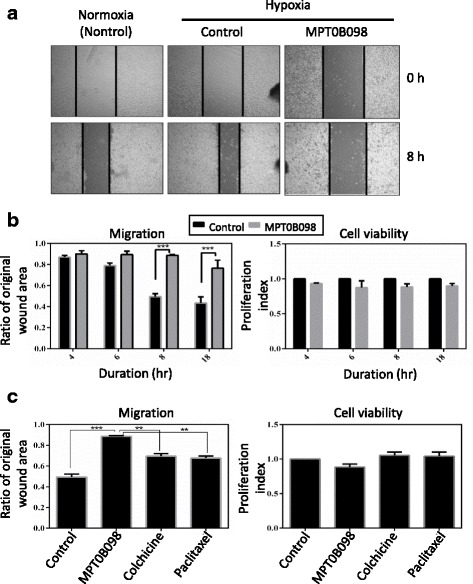
MPT0B098 inhibits hypoxia-induced cell migration in OEC-M1 cells. **a** Effect of MPT0B098 on cell motility. OEC-M1 cells were treated with 0.5-fold IC_50_ of MPT0B098 for 8 h and then cell motility was determined by the wound healing assay. **b** Quantification of cell migration was carried out by measuring the wound area (*left panel*) and calculating cell viability (*right panel*) after cells were treated with 0.5-fold IC_50_ of MPT0B098 for 4, 6, 8, and18 h. **c** Parallel comparison of the effect of MPT0B098 with other microtubule inhibitors, including colchicine and paclitaxel, at the drug concentration of 0.5-fold IC_50_on cell motility (*left panel*) and viability (*right panel*) at a treatment duration of 8 h. Data are represented as mean ± SD in triplicate. **P < 0.01 and ***P < 0.001 for comparison between the control and treatment groups using an unpaired two-tailed Student’s t test

Since EMT is associated with tumor migration, especially under hypoxic conditions [4, 24], we investigated whether MPT0B098 inhibits the EMT program, which then leads to suppression of the migratory capability of OEC-M1 cells in hypoxia. In the wound healing assay we observed that hypoxia induces greater cell migration compared with normoxia, whereas MPT0B098 inhibits hypoxia-induced cell migration with 8 h treatment in OEC-M1 cells (Fig. 8a). From quantification of the wounded area we found that MPT0B098 effectively inhibited hypoxic cell migration in a time-dependent manner (Fig. 8b, *left panel*), without impairing cell viability (Fig. 8b, *right panel*), compared with control cells with a treatment duration of 4–18 h. Moreover, we compared the antimigratory effect of MPT0B098 with other microtubule inhibitors, including colchicine and paclitaxel, and found that the wound healing inhibitory activity of MTP0B098 was significantly higher than colchicine and paclitaxel at a drug concentration of 0.5-fold IC_50_, under hypoxic conditions (Fig. 8c).

## Discussion

Microtubule-targeting drugs, such as taxotere, epothilone B, discodermolide, vincristine, 2-methoxyestradiol, and colchicine, can downregulate the expression of HIF-1α protein but not HIF-1α mRNA [25]. MPT0B098, a novel indoline-sulfonamide-based microtubule inhibitor, can destabilize HIF-1α mRNA in hypoxia by inhibiting the translocation of HuR from the nucleus to cytoplasm [12]. This ability distinguishes MPT0B098 from other microtubule inhibitors. MPT0B098 is effective against a panel of human cancer cell lines, regardless of the p-gp170/MDR status [10–12]. Because MPT0B098 has a unique ability to modulate HIF-1α, we proposed that MPT0B098 also suppresses hypoxia-induced malignant transformation.

Hypoxia can induce EMT and enhance the migration and invasion ability of tumor cells [26]. EMT is a key step for tumor metastasis. Hypoxia leads to characteristic changes in cell morphology, causing a mesenchymal-like phenotype, a distinct characteristic of the EMT process [4]. In this study, we selected a highly invasive human HNSCC cell line, OEC-M1 [27], to clarify the role of MPT0B098 in EMT regulation. We observed that MPT0B098 could inhibit expression of HIF-1α protein and the hypoxia-induced mesenchymal-like phenotype in OEC-M1 cells (Fig. 2c). In addition, this compound was more effective, as indicated by low hypoxia-induced drug resistance, than colchicine and paclitaxel in inhibitingOEC-M1 cell growth under hypoxic conditions (Fig. 1c). As determined by expression of the two mesenchymal markers vimentin and N-cadherin, we noted that MPT0B098 was more potent in inhibiting hypoxia-induced vimentin and N-cadherin upregulation than colchicine and paclitaxel in OEC-M1 cells (Fig. 3). These results suggest that MPT0B098 is distinct from other clinically used microtubule inhibitors in its inhibition of hypoxia-induced EMT. Unlike the classic reversal of the EMT phenomenon, E-cadherin protein was slightly suppressed by MPT0B098 (Fig. 3a and 3b). Cadherins have been reported to regulate the organization and dynamics of microtubules. This behavior may also affect cadherin biology through microtubule-based vesicular traffic [28]. Thus, the interplay between E-cadherin and microtubules may be disrupted by the inhibition of microtubule polymerization under MPT0B098 treatment. The underlying mechanisms merit further investigation.

Molecular reprogramming occurring during EMT is triggered and orchestrated by various EMT-activating transcription factors, including Twist and SNAI2/Slug [16]. Yang et al. found that HIF-1α regulated the expression of Twist by binding directly to the hypoxia-response element in the Twist proximal promoter. In addition, silencing of Twist in HIF-1α-overexpressing or hypoxic cells reversed EMT and metastatic phenotypes [29]. Cheng et al. reported that treatment with HIF-1α siRNA diminished the upregulation of SNAI2/Slug expression [30]. Because MPT0B098 is effective in suppressing HIF-1α expression, we investigated the effect of MPT0B098 on modulation of Twist and SNAI2/Slug and found that the expression levels of hypoxia-induced Twist and SNAI2/Slug were significantly decreased in a concentration-dependent manner when cells were treated with various concentrations of MPT0B098 (Fig. 4).

Transcription program switching in EMT is induced by signaling pathways mediated by TGF-β, bone morphogenetic protein, Wnt-β-catenin, Notch, Hedgehog, and receptor tyrosine kinases. These pathways are activated by various dynamic stimuli from the local microenvironment. Of note, TGF-β signaling has a dominant role in EMT [22, 30–32]. Here, we demonstrated that MPT0B098 inhibits hypoxia-induced EMT by downregulating the expression levels of TGF-β mRNA (Fig. 7a) and protein (Fig. 7b). We previously demonstrated that MPT0B098 inhibited HuR translocation to the cytoplasm [12]. HuR, an mRNA-stabilizing protein, shuttles between the nucleus and cytoplasm through several export pathways. Mobilizing HuR from the nucleus to the cytoplasm leads to maintenance of the stability of HuR-mediated target mRNAs. Increased stability of TGF-β mRNA by HuR promotes EMT in pancreatic cancer [33]; therefore, whether MPT0B098 affect the stability of TGF-β mRNA through HuR dependent or independent mechanisms which needs to be further investigated.

**Fig. 9:**
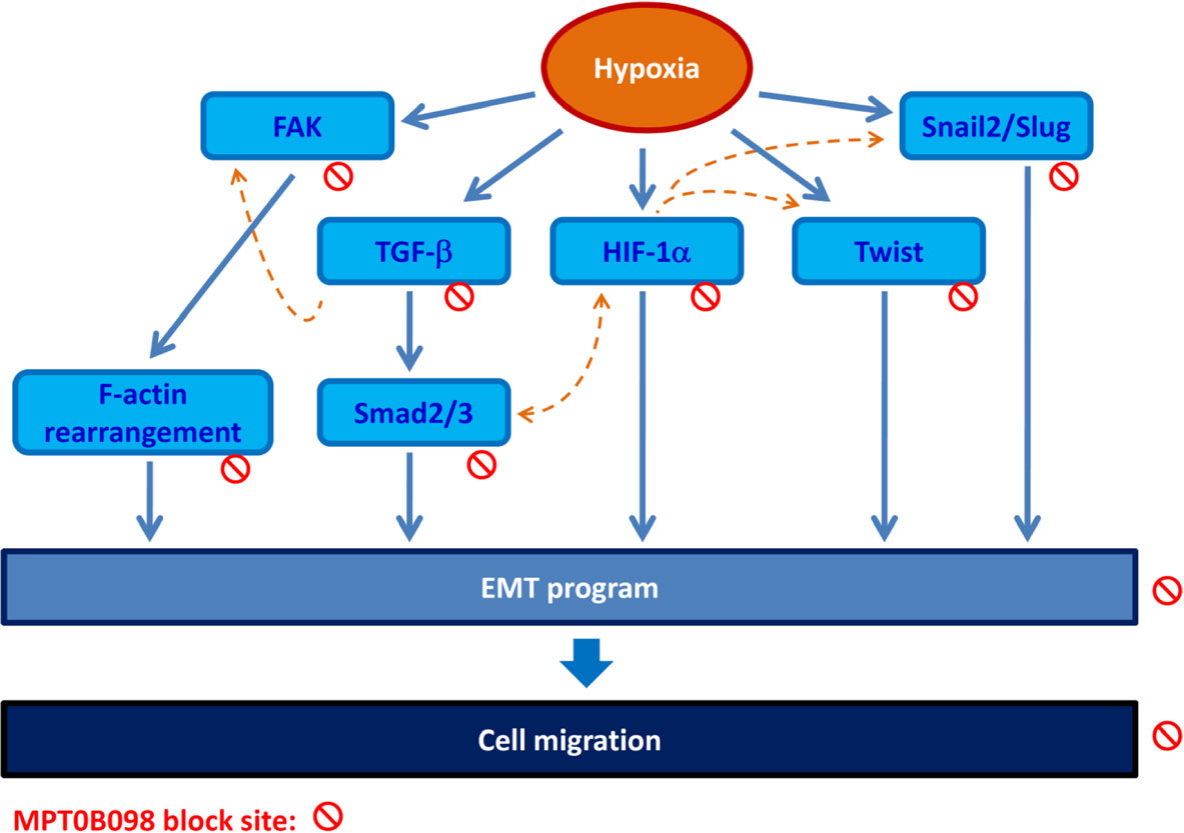
Proposed pathway of MPT0B098-mediated EMT suppression. Blue solid line: proposed working models; orange dotted line: literature reported

TGF-β signaling toward EMT is mediated by both Smad-dependent and -independent pathways. The Smad pathway is unique to TGF-β signaling [34]. Our further evaluation demonstrated that MPT0B098 inhibited hypoxia-induced EMT by blocking TGF-β-dependent Smad signaling (Fig. 6). Because TGF-β signaling occurs not only in hypoxia but also in normoxia, we found that MPT0B098 decreased the expression of TGF-β protein under normoxic conditions (Fig. 7c), suggesting that the inhibitory effect of MPT0B098 on TGF-β signaling was not restricted to the hypoxic condition. Zhang et al. reported that exposure of cells to hypoxia resulted in phosphorylation of Smad2 and Smad3 as well as stimulation of transcriptional activities of HIF-1α and upregulation of *TGF-β2* expression, suggesting that autocrine regulation of TGF-β2 production in hypoxia may involve crosstalk between Smad3 and HIF-1α signaling pathways [35]. The interplay between each molecule in response to MPT0B098 needs further elucidation. In addition to TGF-β/Smad signaling, Cicchini et al. reported that TGF-β induces a Src-dependent activation of FAK protein [36]. The results shown in Fig. 5b show that MPT0B098 significantly suppressed hypoxia-induced FAK phosphorylation. Because FAK is a critical modulator in regulating actin cytoskeleton organization [19–21], we further observed that MPT0B098 inhibited hypoxia-induced expression of the stress fiber pattern and membrane localization of F-actin (Fig. 5a). Accordingly, we proposed that MPT0B098 inhibits hypoxia-induced EMT in HNSCC by (1) suppressing HIF-1α expression, (2) inhibiting the EMT-activating transcription factors Twist and SNAI2/Slug, (3) blocking TGF-β/Smad signaling, and (4) interfering with FAK-mediated actin cytoskeleton rearrangement (Fig. 9). Further evaluation to clarify the interplay between MPT0B098 and the particular molecules is warranted.

## Conclusions

Taken together, we demonstrated for the first time that the novel microtubule Inhibitor MPT0B098 inhibits hypoxia-induced EMT in HNSCC through modulation of HIF-1α, Twist, SNAI2/Slug, TGF-β/Smad signaling, and FAK/actin cytoskeleton rearrangement. The results presented here may provide novel insight into the mechanism of action of microtubule inhibitors for inhibiting EMT. MPT0B098 has great potential for clinical treatment of hypoxic tumors and merits further investigation.

## Additional file

Additional file 1: Supplemental materials. (DOCX 739 kb)

## Abbreviations

EMT: *epithelial-mesenchymal transition*
FAK: focal adhesion kinase
GAPDH: glyceraldehyde 3-phosphate dehydrogenase
HIF-1α: hypoxia-induced factor 1 alpha
HNSCC: head and neck squamous cell carcinoma
HuR: human antigen R
TGF-β: transforming growth factor beta
