# Supplementary material for: Novel microtubule inhibitor MPT0B098 inhibits hypoxia-induced epithelial-to-mesenchymal transition in head and neck squamous cell carcinoma
Source: J Biomed Sci. 2018 Mar 28;25:28. doi: 10.1186/s12929-018-0432-6 (PMC5875002; doi:10.1186/s12929-018-0432-6)
Supplement: Supplementary file 1 — Supplemental materials. (DOCX 739 kb) [file 12929_2018_432_MOESM1_ESM.docx]

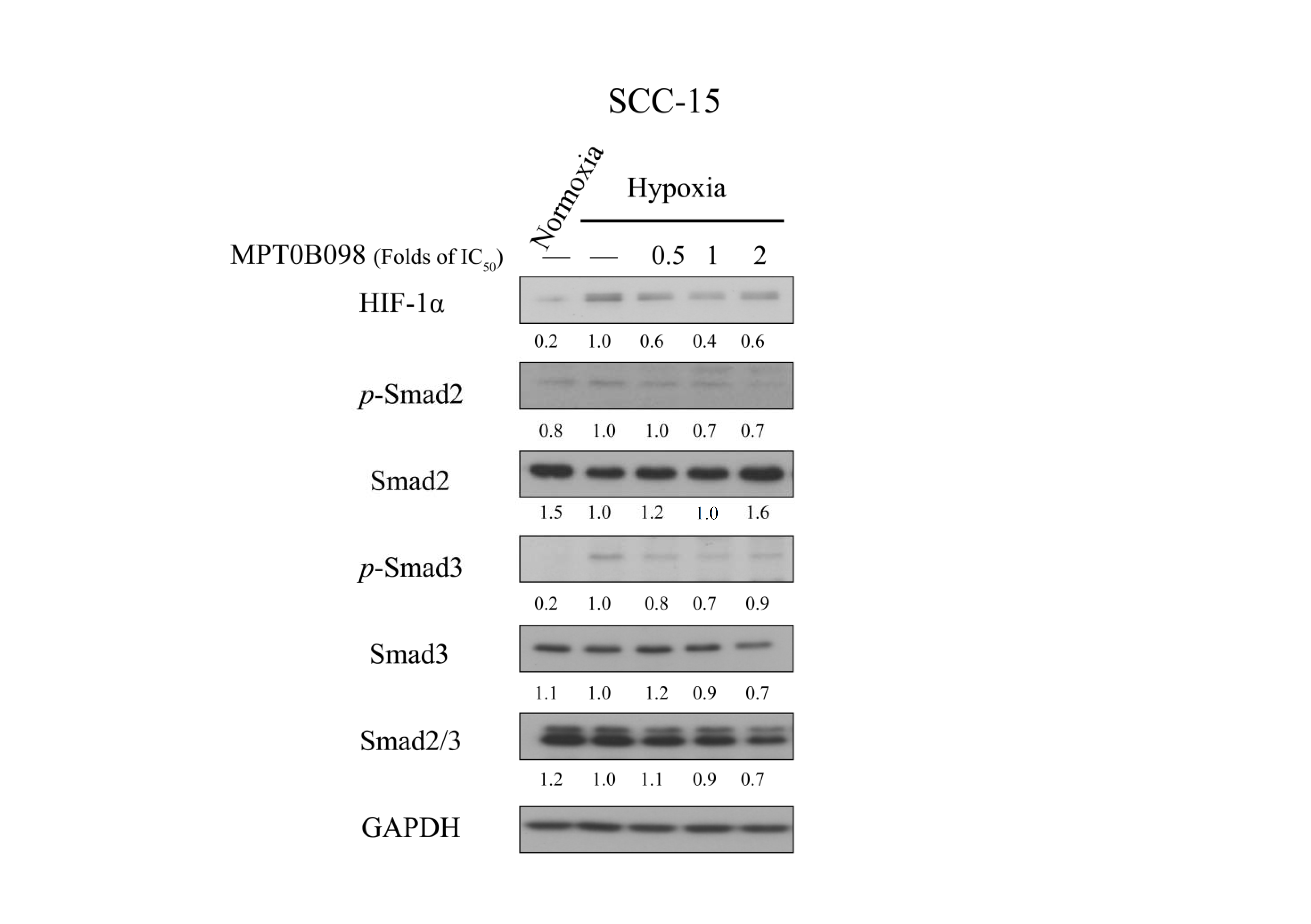


**Figure S1. MPT0B098 suppresses hypoxia-induced HIF-1α expression and Smad phosphorylation in human oral cancer SCC-15 cell line**. SCC-15 cells were treated with MPT0B098 under hypoxia for the indicated concentrations. After incubation for 36 hours, cell lysates were prepared for western blot analysis and probed with HIF-1α, *phospho*-smad2, smad2, *phospho*-smad3, smad, smad2/3, and GAPDH was used as an internal control. Cells in normoxia condition were used as control.
